# Ivanilda (Vanny) Reis: encouraging physical activity and sport for everyone

**DOI:** 10.2471/BLT.24.030624

**Published:** 2024-06-01

## Abstract

Vanny Reis talks to Gary Humphreys about the transformative power of sport and physical activity in improving health and well-being throughout people’s lives.

*Q: You grew up on an island in the Republic of Cabo Verde. To what extent was sport a part of your upbringing?*

A: I grew up in a small village called Ribeira das Patas on the island of Santo Antão, and my earliest memories are of walking. I walked everywhere, climbing hills to visit friends and family, including one of my grandmothers who used to live in a very remote area that is still only accessible by walking. So, I grew up walking. And climbing trees, of course. There were trees everywhere.

*Q: Did you play sport at school?*

A: I did. Because I was tall, I was encouraged to play basketball by one of my teachers at school and was selected to play for the national team when I was 17 years old, but – and this is interesting, I think – I didn’t continue because I didn’t feel included. And I needed special shoes to play on the basketball court. So, I joined the handball team instead. It was what I was able to afford, and it also connected me with kids from similar backgrounds. I would play handball outdoors with other kids, even barefoot. We didn’t need a special court, or hoops or special shoes, and it was fantastic! That was my first taste of sport for all. I was eventually scouted (selected by a talent spotter) and invited to play handball for Batuque Futebol Clube in Mindelo. That team became my whole life. I mean, I grew up with two sisters and two brothers, but everything I did, all my social activities were around my team.

*Q: How did you get from your island to studying physical education at university in Brazil?*

A: I was lucky enough to win a scholarship and to have a mother who was determined for me to get an education. All I had to pay for was the plane ticket, which I did with money I had earned working as a receptionist. I left Cabo Verde thinking I was going to Campinas, a city in southeast Brazil not far from São Paulo, but ended up in a very remote part of the country. It was quite challenging at first, but I learned so much, especially in the fourth year when I specialized in sport and health. I came to see that sport and physical activity generally, impacts more than physical health. It brings people together and it empowers, and it is for everybody, all ages.

“Sport […] impacts more than physical health.”

*Q: Was there any particular moment when that fully inclusive vision came into focus for you?*

A: There were lots of them, but I do remember – and this was in my second year at university – I started working as a trainee in a gym. I worked as a personal trainer, helping people with their workouts, and I was assigned to work with an 80-year-old man. He was at the gym every morning at 6 a.m. He was very strong and independent, knew how every machine worked, and only required minimal supervision. But I could see that the sessions provided him with a routine, structure, social interaction. This experience spurred my interest in working with older adults, and I started teaching hydro-gymnastics classes, which I found incredibly rewarding. It made me think about the accessibility of such programmes in my own country. I asked myself where my grandmother would go if she wanted to exercise, who would assist her, and how she could exercise safely, considering this might be her only social interaction outside of church activities.

*Q: How did those experiences inform your development when you returned to your country?*

A: (laughing) First, it made it impossible to stay at home! I’d grown too independent! Three weeks after I got back, I moved to Praia, the capital, and got a job at a gym. I was so eager to help people, teach them how to exercise, manage the gym, and apply all the experience I had gained. And it was a fascinating time. The whole gym culture was changing, from focusing primarily on muscle gain to incorporating more diverse fitness activities such as yoga and Pilates. And the doors were also opening to the surrounding communities. I remember one day someone walked into the gym with an invitation to celebrate municipality day, inviting all gyms to participate and lead classes. It was an amazing experience, similar to what I had experienced in Brazil, where gyms would collaborate for public events on Sundays. That was my first time participating in such an event outdoors, co-leading a class with another instructor in front of such a diverse group of people. It was a perfect example of inclusivity and accessibility.

*Q: And then, in 2011, you entered a beauty pageant. Was that a new departure for you?*

A: (laughing) Not so much! Even before university, I had participated in fashion and beauty pageants. So, for me it was just a natural extension of those activities. Anyway, I was lucky enough to win, and ended up representing Cabo Verde in the Miss West Africa International pageant and was subsequently crowned Miss West Africa International. That opened a lot of doors for me, and provided a platform to advocate for health and physical activity. I also promoted the cause of natural beauty, empowering women and girls to embrace their natural selves – their hair, the colour of their skin. I also got to meet a lot of high-profile people, including the President of Cabo Verde, Jorge Carlos Fonseca, with whom I discussed the projects I wanted to support during my reign.

*Q: What was your focus at that time?*

A: I was particularly interested in supporting physical activity programmes for the general population, including the elderly. I really felt that sport should be for everyone, not just athletes, and I took every opportunity to get that idea across whenever I did events like product launches or openings. Of course, I knew that I wasn’t going to be a beauty queen forever, and I became an active member of the Global Shapers Community, an initiative of the World Economic Forum that seeks to empower and connect young leaders to help them shape decision-making processes and drive positive change in their communities and the world. I was also active with the youth association WEDOCARE in Praia. Then in 2016, I won a Mandela Washington Fellowship for Young African Leaders (part of the Young African Leaders Initiative run by the Government of the United States of America) and studied business and entrepreneurship in Chicago, where I was recognized as a young African leader by President Obama himself.

*Q: How did you come to work with your country’s sports ministry?*

A: I was invited to join the ministry as an advisor, and was asked to set up a national programme to encourage physical activity. It was the first time I had worked on public policy and I was trying to bring about a population-level change. So, it was quite daunting, but fascinating too, and I really threw myself into it, reading as much as I could. That included WHO’s *Global recommendations on physical activity for health w*hich were published in 2010, and have since been replaced by the guidance published in 2020. The end result of all that reflection and collaboration with my colleagues was a public health initiative designed to encourage physical activity among the general population, which we called MexiMexê.

“Sport should be for everyone, not just athletes.”

*Q: What does MexiMexê mean?*

A: MexiMexê is Cabo Verdean Creole for "move move", and the programme aims to make physical activity a part of everybody’s daily routine for people of all ages. It also seeks to engage different communities through organized sports and activity sessions accessible to everyone, regardless of their fitness level. Typically, the programme is implemented through community-based activities such as organized walks and runs. There are also free public exercise classes such as aerobics, Zumba, and traditional dance offered in public spaces like parks and squares. The programme also focuses on educating the public about the benefits of physical activity, and is integrated into the school curriculum to instill the habit of exercise among children from a young age. You clearly cannot start too early with this. After MexiMexê, I went on to work with the Cabo Verde Institute of Youth and Sport, overseeing youth affairs and sports development in the country, and I saw what the challenges were and what a difference sport can make in the life of children as it did in my own childhood. And as I said before, the benefits go way beyond physical fitness.

*Q: The MexiMexê initiative was highlighted as a model of good practice by WHO in 2019. What has been its impact?*

A: The programme has made a significant contribution to raising public awareness about the importance of physical activity and to increasing participation in sport. However, as in most countries, the challenges faced in Cabo Verde are considerable. Many of them – such as increasingly sedentary lifestyles and the consumption of ultra-processed convenience foods – are linked to increasing urbanization. I am currently focusing on the nutrition side of the challenge as a partner in a startup making frozen vegan food and promoting healthy eating through accessible products. However, changing what we eat and the way we eat will only take us so far. We also need to think about the extent to which we move our bodies and ways in which we can encourage that movement. Urban planners have a key role to play here, for example, by building bike lanes and pedestrian-friendly streets, or through the creation and maintenance of attractive public spaces and parks. Implementing traffic calming measures to create safer environments for pedestrians and cyclists, can also make a huge difference. I recently moved to Amsterdam in the Netherlands to be with my partner, and I’m happy to see that so many of the things that I have long advocated for in urban environments, such as green spaces and plentiful opportunities for physical activity – people here cycle everywhere – are already in place. I would like to see the same in cities everywhere.

